# *Psoralea corylifolia* L. Seed Extract Attenuates Diabetic Nephropathy by Inhibiting Renal Fibrosis and Apoptosis in Streptozotocin-Induced Diabetic Mice

**DOI:** 10.3390/nu9080828

**Published:** 2017-08-02

**Authors:** Eunhui Seo, Hwansu Kang, Yoon Sin Oh, Hee-Sook Jun

**Affiliations:** 1 College of Pharmacy and Gachon Institute of Pharmaceutical Science, Gachon University, Incheon 21936, Korea; eunhuiseo@gachon.ac.kr (E.S.); hwansu83@naver.com (H.K.); 2 Lee Gil Ya Cancer and Diabetes Institute, Gachon University, Incheon 21999, Korea; 3 Department of Food and Nutrition, Eulji University, Seongnam 13135, Korea; ysoh@eulji.ac.kr; 4 Gachon Medical Research Institute, Gil Hospital, Incheon 21565, Korea

**Keywords:** diabetic nephropathy, *Psoralea corylifolia* L. seed, renal fibrosis, renal apoptosis

## Abstract

The *Psoralea corylifolia* L. seed (PCS) is a widely used herbal medicine, but its possible effect against diabetic nephropathy has not been studied. To investigate the anti-nephropathic effect of PCS extracts, we performed experiments using a diabetic mouse model and high glucose-treated mesangial cells. Streptozotocin (STZ)-induced diabetic mice were orally administered PCS extract for 8 weeks (500 mg/kg/day). Increased creatinine clearance, urine volume, urine microalbumin, and mesangial expansion were observed in STZ-induced diabetic mice; these were significantly reduced by PCS extract administration. PCS extract significantly reduced fibrosis in the kidney tissue of diabetic mice as evidenced by decreased mRNA expression of collagen type IV-α2, fibronectin, PAI-1, and TGF-β1. In addition, cleaved PARP, an apoptotic gene, was upregulated in the diabetic nephropathy mice, and this was ameliorated after PCS extract treatment. Treatment of high glucose-treated MES-13 cells with isopsoralen and psoralen, major components of PCS extract, also decreased the expression of fibrosis and apoptosis marker genes and increased cell viability. PCS extract exerts protective effects against STZ-induced diabetic nephropathy via anti-fibrotic and anti-apoptotic effects. PCS extract might be a potential pharmacological agent to protect against high glucose-induced renal damage under diabetic conditions.

## 1. Introduction

Diabetic nephropathy is a common microvascular complication in diabetic patients, which leads to high morbidity and mortality throughout the world [1,2]. Diabetic nephropathy is characterized by structural as well as functional abnormalities. Urinary albumin excretion along with extracellular matrix accumulation, basement membrane thickening, mesangial hypertrophy, and glomerular epithelial cell (podocyte) loss within the glomeruli are characteristic pathological features of diabetic nephropathy [3,4].

Especially, changes in glomeruli such as fibrosis and apoptosis of mesangial cells play important roles in the progression of diabetic nephropathy. The hyperglycemic condition induces expression of genes associated with fibrosis, such as transforming growth factor-β (TGF-β), fibronectin, collagen type IV, α2 (Col4a2), and plasminogen activator inhibitor-1 (PAI-1) [5,6]. Moreover, activation of apoptotic programs such as nuclear condensation, caspase activation, and release of cytochrome c from mitochondria are observed in high glucose-treated mesangial cells [7,8].

Intensive blood glucose control and anti-hypertensive agents such as angiotensin converting enzyme inhibitors and angiotensin receptor-1 antagonists are currently the most effective treatments for progressive diabetic nephropathy [9]. However, there are several limitations to these drugs, such as patient resistance and heart or kidney failure [10,11]. Therefore, the development of new drugs or adjuvants that act on various components of diabetic nephropathy is urgently required.

*Psoralea corylifolia* L. seed (PCS), commonly known as “Boh-Gol-Zhee” in Korea, has been used in herbal and traditional medicine for various diseases including diabetes, cancer, inflammatory disease, neurodegenerative disease, and kidney disease [12]. Six compounds, bakuchiol, psoralen, isopsoralen, corylifolin, corylin, and psoralidin, are the major components of PCS extract [13]. Among these, bakuchiol, which is a meroterpene, and psoralen and isopsoralen, which are coumarins, have been widely studied, and their health benefits regarding anti-oxidant, anti-tumor, and estrogenic activity have been identified [14,15,16,17].

Previously, we found that PCS extract shows protective effects on oxidative stress-induced pancreatic beta cell apoptosis [18] and hepatic damage [19]. Furthermore, PCS extract shows promise as an anti-obesity agent in a high fat diet-induced obesity model [20], suggesting that PCS extracts might also have ameliorative effects on diabetic nephropathy. Therefore, we investigated whether PCS extracts have beneficial effects on diabetic nephropathy in a streptozotocin (STZ)-induced type 1 diabetic mouse model and investigated the mechanisms involved in high glucose-treated glomerular cells.

## 2. Materials and Methods

### 2.1. Reagents

Dulbecco modified eagle medium (DMEM), Ham’s F-12 (F-12) medium, and fetal bovine serum (FBS) were purchased from Gibco BRL (Grand Island, NY, USA). Bakuchiol was purchased from Enzo Life Sciences Inc. (Farmingdale, NY, USA). Antibodies against poly (ADP-ribose) polymerase (PARP), B-cell lymphoma (Bcl)-2, Bcl-2-associated death promoter (Bad), and phospho-Bad were obtained from Cell Signaling Technology (Beverly, MA, USA). Antibodies against beta-actin and horseradish peroxidase-conjugated secondary antibodies were obtained from Santa Cruz Biotechnology Inc. (Santa Cruz, CA, USA). Streptozotocin (STZ), losartan potassium, psoralen, and isopsoralen were obtained from Sigma-Aldrich (St. Louis, MO, USA).

### 2.2. Preparation of PCS Extract

PCS was purchased from an oriental drug store (Kwang Myung Dang Co., Ulsan, Korea), and the extract was prepared by the standard procedure as described previously [18,19]. In summary, 300 grams of dry seed were ground into small pieces and then extracted twice with distilled water under reflux. The combined water extracts were evaporated in vacuo and finally yielded 61.92 g of a dark brown residue.

### 2.3. Animals

Six-week-old male C57BL/6 mice were supplied by the Orient Bio Inc (Seongnam, Gyeonggi-do, Korea). Animals were maintained at animal facilities at the Lee Gil Ya Cancer and Diabetes Institute, Gachon University of Medicine and Science, under a 12-h light, 12-h dark photoperiod. All animal experiments were carried out under a protocol approved by the Institutional Animal Care and Use Committee (LCDI-2012-0029) at Lee Gil Ya Cancer and Diabetes Institute, Gachon University. After a week of adaptation, mice were injected intraperitoneally with 50 mg/kg/day STZ for five consecutive days. Age-matched control mice received an equal volume of vehicle. After one week after the fifth STZ injection, blood glucose levels were checked, and mice with blood glucose levels over 300 mg/dL were used for experiments. STZ-induced diabetic mice were treated orally with PCS extract (500 mg/kg/day) or vehicle (daily for 8 weeks), as described previously [18,20]. Losartan potassium (Sigma-Aldrich) was orally administered (10 mg/kg/day) for 8 weeks as a positive control.

### 2.4. Periodic Acid-Schiff (PAS) Staining

After 10% formalin fixation, 4 μm sections cut from paraffin-embedded kidney samples were stained with PAS. Thirty glomeruli were randomly selected from each mouse for the PAS analysis using Image J software (version 1.48q, National Institutes of Health, Bethesda, MD, USA). The mesangial matrix index is the ratio of mesangial matrix area divided by the tuft area.

### 2.5. Biochemical Parameters in Blood and Urine

Urinary and serum parameters, such as creatinine, urea nitrogen, total protein, and microalbumin, were measured by an immunoturbidimetric method using an AU680 automated chemistry analyzer (Beckman Coulter, Inc., Brea, CA, USA). Creatinine clearance was calculated using following calculation formula. Creatinine clearance = urine volume (mL/min) × urine creatinine/serum creatinine

### 2.6. Murine Mesangial Cell Culture

SV40-transformed murine glomerular mesangial cells (MES-13) were obtained from American Type Culture Collection (ATCC, Rockville, MD, USA) and maintained in a 3:1 mixture of DMEM and F-12 medium containing FBS (5%), penicillin (100 U/mL), streptomycin (100 μg/mL), HEPES (14 mM), and glucose (5.5 mM) at 37 °C in an atmosphere containing 5% CO_2_–95% air. To test the response of high glucose concentration, the cells were maintained in 25 mM glucose-containing medium for 24 h. Cells were cultured in the presence of 5.5 mM glucose and 19.4 mM mannitol as an osmotic control. The number of viable cells was determined using Cell Counting Kit-8 (CCK-8) assay kit (Dojindo Laboratories, Kumamoto, Japan), which measures dehydrogenase activities in cells.

### 2.7. Western Blot Analysis

Cells were lysed with mammalian protein extraction buffer (GE Healthcare, Milwaukee, WI, USA) containing a protease inhibitor and phosphatase inhibitor cocktail (Sigma-Aldrich). A standard amount of protein was resolved by sodium dodecyl sulfate polyacrylamide gel electrophoresis, transferred onto membranes, and blocked [20]. The membranes were incubated with specific primary antibodies and horseradish peroxidase-conjugated secondary antibodies. Chemiluminescence was detected on LAS-4000 (Fuji Film, Tokyo, Japan) using Immobilon Western Chemiluminescent HRP Substrate (Millipore, St. Charles, MO, USA). The protein bands obtained by western blotting were analyzed using ImageJ (National Institutes of Health, Bethesda, MD, USA) software for Windows.

### 2.8. Quantitative Real-Time RT-PCR (qRT-PCR) Analysis

Total RNA was extracted using TRIZOL reagent (Invitrogen Corp., Carlsbad, CA, USA), and cDNA was synthesized using a PrimeScript 1st strand cDNA synthesis kit (Takara Bio Inc., Kyoto, Japan). qRT-PCR was performed as previously described [20]. The relative copy number was calculated using the threshold crossing point (Ct) as calculated by ΔΔ Ct. Primer sequences were as follows: 5′-TGGAGAGCACCAAGACAGACA-3′ and 5′-TGCCGGAGTCGACAATGAT-3′ for mouse cyclophlin; 5′-TGACGATGGGAAGACCTACCA-3′ and 5′-GGAACAAATGGCTCCGAGATAT -3′ for mouse fibronectin; 5′-TCAATGACTGGGTGGAAAGG-3′ and 5′-AGGCGTGTCAGCTCGTCTAC-3′ for mouse PAI-1; 5′-GCAAAAGGTCAGGATCGAGGTA-3′ and 5′-GTGCCGAACCACAAAGAGAAAG-3′ for mouse Col4a2 and 5′-GCAGTGGCTGAACCAAGGA-3′ and 5′-AGCAGTGAGCGCTGAATCG-3′ for mouse TGF-β1.

### 2.9. Statistical Analyses

All data are expressed as mean ± standard error of at least three independent experiments. Data were analyzed using Analysis of Variance followed by post-hoc analysis using the Tukey range test (SPSS 10.0 statistical software). The *p*-values less than 0.05 were considered to be statistically significant.

## 3. Results

### 3.1. PCS extract Treatment Improved Renal Function in STZ-Induced Diabetic Mice

**Figure 1:**
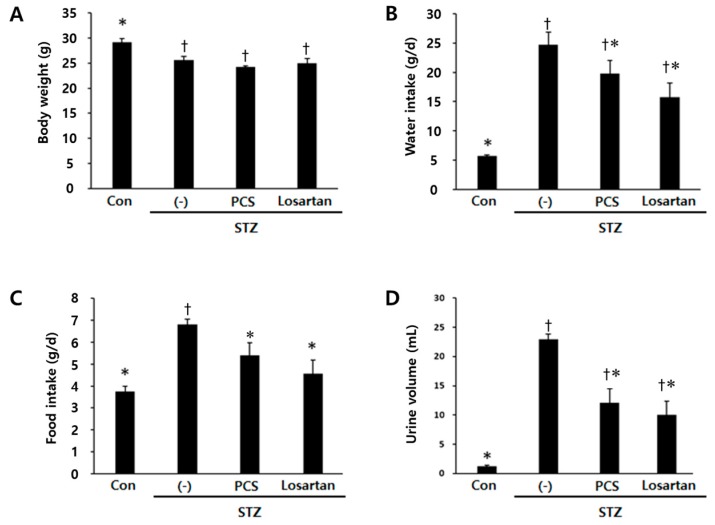
Body weight, food and water intake, and urine volume in PCS extract-treated, STZ-induced diabetic mice. Mice were injected with vehicle (Con) or STZ (50 mg/kg/day) for five consecutive days. STZ-induced diabetic mice (above 300 mg/dL blood glucose) were treated with vehicle (-), PCS extract (500 mg/kg/day), or losartan potassium (10 mg/kg/day) as a positive control for 8 weeks (*n* = 8–11/group). (**A**) Body weight after 8 weeks of treatment; (**B**) average water intake over 8 weeks; (**C**) average food intake over 8 weeks; and (**D**) urine volume for 24 h after 8 weeks of PCS extract treatment; †, *p* < 0.05 vs. Con mice. *, *p* < 0.05 vs. (-)/STZ mice.

**Figure 2:**
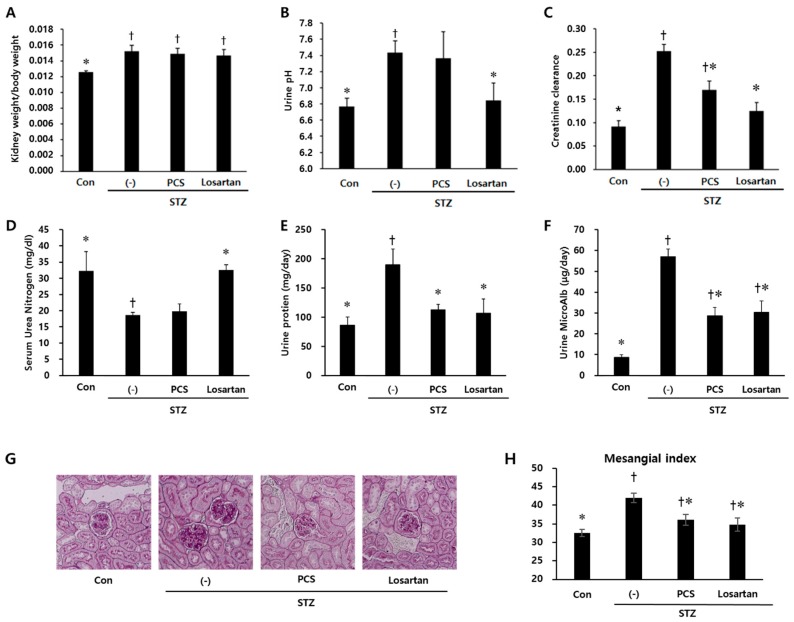
Parameters of renal function in PCS extract-treated, STZ-induced diabetic mice. Mice were treated as described in Figure 1 (*n* = 8–11/group). (**A**) The ratio of kidney weight/body weight; (**B**) Urine pH; (**C**) Creatinine clearance; (**D**) Serum urea nitrogen; (**E**) Urinary protein; (**F**) Urinary microalbumin; (**G**) Representative photomicrographs (original magnification, 200×) of PAS staining of kidney sections; and (**H**) Mesangial matrix index after 8 weeks of PCS extract treatment. †, *p* < 0.05 vs. Con mice. *, *p* < 0.05 vs. (-)/STZ mice.

Body weight was significantly reduced in STZ-induced diabetic mice compared with control mice and PCS extract treatment did not recover the reduced body weight (Figure 1A). Water intake, food intake and urine volume were increased in STZ-induced diabetic mice compared with control mice. After PCS extract administration, water and food intake were significantly reduced (Figure 1B,C). Furthermore, the increased urine volume in STZ-induced diabetic mice was significantly reduced in PCS extract-treated mice (Figure 1D). Treatment with losartan potassium, which was used as positive control, significantly reduced water intake, food intake, and urine volume compared with vehicle-treated diabetic mice (Figure 1). To investigate whether renal function was improved by PCS extract administration, biochemical parameters in serum/urine and pathological changes were analyzed. Kidney weight and urine pH were significantly increased in STZ-induced diabetic mice compared to control mice, but PCS extract treatment in STZ-diabetic mice did not change these parameters (Figure 2A,B), but the increased creatinine clearance, urine protein, and urine microalbumin seen in STZ-induced diabetic mice were significantly reduced by PCS extract treatment (Figure 2C–F). Renal histological analysis showed that the increase of the mesangial matrix index in STZ-injected mice was significantly ameliorated by PCS extract treatment, and the effect was similar to losartan treatment (Figure 2G,H).

### 3.2. PCS Extract Inhibited the Expression of Genes Related to Renal Fibrosis and Apoptosis in STZ-Induced Diabetic Mice

**Figure 3:**
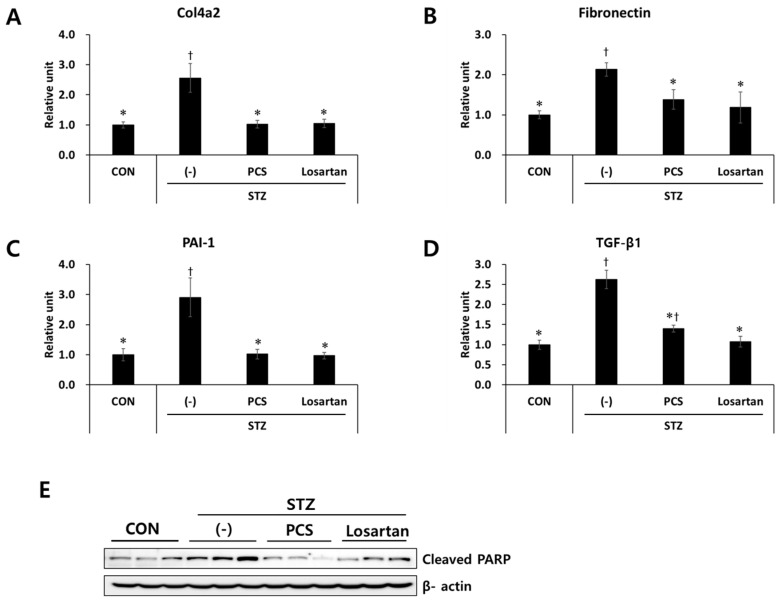
Effect of PCS extract on the expression of fibrosis-related genes in PCS extract-treated, STZ-induced diabetic mice. Mice were treated as described in Figure 1 (*n* = 8–11/group). After 8 weeks of PCS extract treatment, total RNA was extracted from the kidney tissue and qRT-PCR analysis was performed for: (**A**) Col4a2; (**B**) fibronectin; (**C**) PAI-1; and (**D**) TGF-β1. The mRNA levels were normalized with those of cyclophilin. (**E**) Protein was extracted and western blotting analysis was carried out for cleaved PARP. †, *p* < 0.05 vs. CON mice. *, *p* < 0.05 vs. (-)/STZ mice.

As we found that PCS extract treatment reduced mesangial expansion in diabetic mice, we next measured the expression levels of fibrosis-related genes in the kidney tissue by q-RT-PCR analysis. mRNA expression levels of fibrosis-related genes such as Col4a2, fibronectin, PAI-1, and TGF-β1 were significantly increased in STZ-induced diabetic mice, and these increases were inhibited by PCS extract treatment (Figure 3A–D). As apoptotic cell death is correlated with diabetic nephropathy [21], we examined expression level of an apoptotic marker in the kidney tissue of diabetic mice with or without PCS extract treatment. As shown in Figure 3E, cleaved PARP expression was increased in vehicle-treated diabetic mice compared with nondiabetic mice, and PCS extract administration significantly reduced STZ-induced PARP expression (Figure 3E). Losartan treatment also reduced the expression level of fibrosis related genes and cleaved PARP in diabetic mice (Figure 3A–E).

### 3.3. PCS Extract or Its Components Protected Cells from High Glucose-Induced Apoptosis in Mesangial Cells

**Figure 4:**
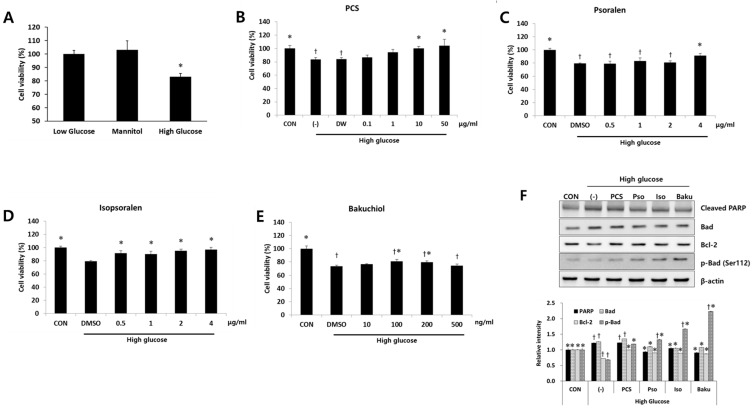
Effect of PCS extract and its components on viability and apoptosis in high glucose-treated mesangial cells. (**A**) SV40 MES13 cells were cultured with low glucose (5.5 mM), 5.5 mM glucose and 19.4 mM mannitol, or high glucose (25 mM) for 24 h and cell viability were determined by CCK-8 assay. *, *p* < 0.05 vs. Low glucose. SV40 MES-13 cells were treated with high glucose concentration (25 mM) with or without (-) various concentrations of PCS extract or its components for 24 h. Cells cultured in the presence of 5 mM glucose without any treatment were used as a control (CON). Cell viability after treatment with: (**B**) PCS extract; (**C**) Psoralen; (**D**) Isopsoralen; or (**E**) Bakuchiol were determined by CCK-8 assay. †, *p* < 0.05 vs. CON. *, *p* < 0.05 vs. (-)/High glucose. (**F**) Protein was prepared after treatment with PCS extract (50 μg/mL), psoralen (4 μg/mL), isopsoralen (4 μg/mL), or bakuchiol (200 ng/mL), and western blotting analysis was carried out for cleaved PARP, Bad, Bcl-2, and p-Bad (Ser112). Actin was used as a loading control. Corresponding densitometric quantification of western blotting analysis is presented at the bottom. The results shown represent the mean ± SEM from three independent duplicate experiments. †, *p* < 0.05 vs. CON. *, *p* < 0.05 vs. (-)/High glucose.

To investigate the mechanisms and specific components of PCS extract responsible for the protective effect in the kidney of diabetic mice, in vitro studies were performed using mouse mesangial MES-13 cells. Bakuchiol, psoralen, and isopsoralen are known to be major components of PCS [13], and these are detected in the serum of the PCS extract-treated mice [18]. Therefore, we investigated whether these compounds protect MES-13 cells from high glucose-induced apoptosis using previously determined dose ranges that do not cause cell toxicity (Figure 4 and Figure S2). High glucose treatment significantly reduced cell viability compared with the control, and cell viability was not changed by mannitol treatment (Figure 4A), indicating that the effect is not due to osmotic pressure. Ten or 50 μg/mL of PCS extract recovered cell viability (Figure 4B). Low concentrations (0.5–2 μg/mL) of isopsoralen significantly enhanced cell viability in the presence of high glucose, whereas psoralen was only effective at a higher dose (4 μg/mL) (Figure 4C,D). Bakuchiol treatment (100 or 200 ng/mL) also significantly inhibited high glucose-induced cell death, but the effect was not observed at a higher concentration (500 ng/mL) (Figure 4E), and cell death was observed at concentrations above 500 ng/mL (Figure 4E and Figure S2). We also investigated the expression level of pro-apoptosis proteins after high glucose treatment with or without various compounds. As shown in Figure 4F, high glucose treatment increased the expression levels of cleaved PARP and Bad, and 50 μg/mL PCS extract, 4 μg/mL psoralen, 4 μg/mL isopsoralen, or 200 ng/mL bakuchiol reduced the expression of these proteins. The expression levels of phospho-Bad (ser112) and Bcl-2, pro-survival markers, were decreased by high glucose and increased by PCS extract or its components (Figure 4F).

### 3.4. PCS Extract or Its Components Reduced Expression of Fibrosis-Related Genes in Mesangial Cells

**Figure 5:**
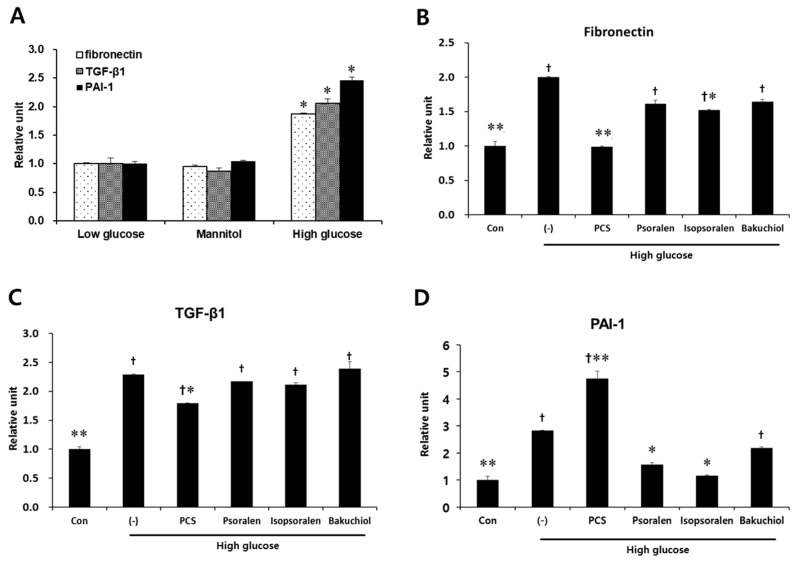
Effect of PCS extract and its components on fibrosis-related gene expression in high glucose-treated mesangial cells. (**A**) SV40 MES13 cells maintained in low glucose (5.5 mM), glucose (5.5 mM) and mannitol (19.4 mM), or high glucose (25 mM) for 72 h. Total RNA was extracted and qRT-PCR analysis was performed. *, *p* < 0.05 vs. Low glucose. (**B**–**D**) SV40 MES-13 cells were treated as described in Figure 4B–F. After 72 h, total RNA was extracted and qRT-PCR analysis was performed for: (**A**) fibronectin; (**B**) PAI-1; and (**C**) TGF-β1. The mRNA levels were normalized with those of cyclophilin. The results shown represent the mean ± SEM from three independent duplicate experiments. †, *p* < 0.05 vs. Con. *, *p* < 0.05 vs. (-)/High glucose. **, *p* < 0.01 vs. (-)/High glucose.

To investigate whether PCS extract also reduced fibrosis in MES-13 cells, we measured the expression level of fibrosis markers such as TGF-β1, fibronectin, and PAI-1 by qRT-PCR analysis. Treatment with mannitol did not change the expression of fibrosis-related genes compared with untreated cells (Figure 5A). Exposure to high glucose alone induced a significant increase in fibronectin, PAI-1, and TGF-β1, and PCS extract significantly reduced the mRNA expression of fibronectin and TGF-β1, but increased the mRNA expression of PAI-1. Treatment with isopsoralen (4 μg/mL) inhibited high glucose-induced fibronectin and PAI-1 mRNA levels, and psoralen treatment (4 μg/mL) inhibited only PAI-1 mRNA. Bakuchiol (200 ng/mL) did not affect the expression of fibrosis-related genes (Figure 5B–D).

## 4. Discussion

Diabetic nephropathy is the most serious complication in diabetes mellitus and causes glomerular fibrosis and impairment of renal function. Currently available drugs, which control blood glucose or blood pressure, have a number of limitations, such as patient resistance and high rates of secondary failure [10]. These lead to the search for alternative therapies from natural products that have low or no side effects and multi-target actions.

PCS extract has generated much interest due to its various biological activities, including anti-inflammation, anti-apoptosis, and anti-hyperglycemic effects [20,22,23], suggesting further study on its effect on diabetic complications such as nephropathy. Therefore, we investigated anti-diabetic nephropathy effects of PCS extract in STZ-induced diabetic mice, which develop renal injury with similarities to human diabetic nephropathy [24].

The increase in urinary albumin excretion, one of the marked renal pathological features in diabetic nephropathy, is caused by mesangial expansion due to accumulation of extracellular matrix components [25]. Our STZ-induced diabetic mice showed an increase in urinary albumin concentration and a corresponding increase in the mesangial matrix index relative to non-diabetic control mice. In addition, the level of creatinine clearance, the most widely used clinical marker of kidney function [26], was higher in diabetic mice than control mice, indicating the presence of diabetic nephropathy with renal hyperfiltration in our STZ-induced diabetic mice. Eight-weeks of PCS extract treatment attenuated the increased albuminuria, creatinine clearance, and mesangial expansion in the glomeruli of STZ-induced diabetic mice. These results demonstrated that PCS extract has potent effects by counteracting mesangial expansion and hyperfiltration in diabetic mice, possibly leading to the amelioration or delay in the development of advanced diabetic renal injury.

We observed that hyperfiltration induced by STZ injection led to a decrease in serum urea nitrogen levels, but administration of PCS extract did not recover this to normal levels. These results show lack of correlation with creatinine clearance and serum urea nitrogen, and this is consistent with another study in STZ-induced diabetic rats fed a high fat diet [27].

Previously, we reported that PCS extract treatment showed anti-hyperglycemic effects via reduced lipid accumulation and reduced inflammation in the liver of mice fed a high fat diet [20]. In the present study, treatment of diabetic mice with PCS extract ameliorated creatinine clearance and mesangial matrix accumulation, but had no impact on glucose homeostasis (Figure S1). These conflicting results might be the result of the different mechanisms used to induce different diabetic mouse models [28]: such as type 1 diabetes (present study) versus type 2 diabetes (previous study). Moreover, effect of PCS extract regarding improvement of renal injury was similar to the losartan-treated group, suggesting that PCS extract might directly improve kidney function by glucose-independent mechanisms.

Several studies have demonstrated that increased TGF-β expression in mesangial cells promotes extracellular matrix accumulation and hypertrophy during progression of diabetic nephropathy [29,30]. Moreover, as increased TGF-β is known to be a potent inducer of Col4a2, fibronectin, and PAI-1 expression [31], we investigated whether PCS extract reduced the expression of these molecules. Treatment with PCS extract inhibited the expression level of fibrosis markers both in the kidney tissue of diabetic mice and in high glucose-treated MES-13 mesangial cells, and also increased cell viability. These results show that the anti-fibrotic effects of PCS extract provided effective protection from high glucose-mediated kidney damage.

Apoptosis plays a pathological role leading to the death of mesangial cells, which is associated with progressive glomerulosclerosis [32,33]. Khera et al. reported that high glucose-mediated TGF-β activation decreases nuclear factor-kB activation and in turn, alters the expression ratio of Bcl-2:Bcl-2-associated X protein favoring caspase-3 activation and increased apoptosis [8]. We also found that expression level of apoptotic makers was increased under diabetic nephropathy conditions, and PCS extract treatment attenuated cleaved PARP and increased Bcl-2 expression and phosphorylation of Bad (Ser-112). Although we did not evaluate the correlation between fibrosis and apoptosis pathways in glucose-treated mesangial cells, these results suggest that inhibition of apoptosis under conditions of high glucose toxicity is an important mechanism of PCS extract in reducing glomerulosclerosis in diabetic nephropathy.

We found that treatment with major compounds of PCS extract (bakuchiol, psoralen, and isopsoralen) inhibited mesangial cell death, but the anti-apoptotic and anti-fibrotic responses varied. Two coumarins, psoralen and isopsoralen, increased cell viability and decreased apoptotic protein expression, and bakuchiol showed anti-apoptotic effects at much lower concentration compared with psoralen or isopsoralen. Similarly, previous studies found that psoralen or isopsoralen inhibits apoptotic cell death in H_2_O_2_-treated INS-1 cells [18] or palmitate-treated PC12 cells [34]. Psoralen or isopsoralen decreased mRNA expression level of fibronectin and PAI-1, suggesting that the effects of PCS extract in diabetic nephropathy was mediated primarily by these compounds. However, total PCS extract treatment significantly increased the expression of PAI-1. PCS extract contains other chemical compounds such as coryfolin, corylin, and 3-hydroxybakuchiol [13], which might have resulted in the increase of PAI-1 expression in apoptotic mesangial cells seen after PCS treatment.

It is known that the treatment of high glucose in mesangial cells increases the level of reactive oxygen species (ROS) [35,36]. Previously, we reported that PCS extract has an anti-oxidative effect in hepatocytes [19] and pancreatic beta cells [18], and this effect is mediated by improvement of mitochondrial function. As oxidative stress plays a role in the pathogenesis of diabetic nephropathy [37], possible involvement of the anti-oxidative effect of PCS extract in the inhibition of diabetic nephropathy will be investigated.

## 5. Conclusions

PCS extract has beneficial effects against diabetic nephropathy via amelioration of high glucose-induced mesangial cell injury. PCS extract treatment decreased glucotoxic effects via anti-apoptotic and anti-fibrotic functions. In addition, isopsoralen and psoralen, components of PCS extract, were effective against high glucose-induced mesangial cell injury. These results provide evidence that PCS extract and its active compounds (isopsoralen and psoralen) may be potential therapeutic agents for reducing glucose-induced mesangial cell death during diabetic nephropathy.

## Supplementary Materials

The following are available online at www.mdpi.com/2072-6643/9/8/828/s1, Figure S1: Blood glucose levels in STZ-induced diabetic mice treated with PCS extract, Figure S2: Cell viability in SV40 MES-13 cells treated with 4 μg/mL of psoralen, isopsoralen or bakuchiol.
