# Supplementary material for: Psoralea corylifolia L. Seed Extract Attenuates Diabetic Nephropathy by Inhibiting Renal Fibrosis and Apoptosis in Streptozotocin-Induced Diabetic Mice
Source: Nutrients. 2017 Aug 2;9(8):828. doi: 10.3390/nu9080828 (PMC5579621; doi:10.3390/nu9080828)
Supplement: Supplementary file 1 [file nutrients-09-00828-s001.zip › nutrients-206352-supplementary.pdf]

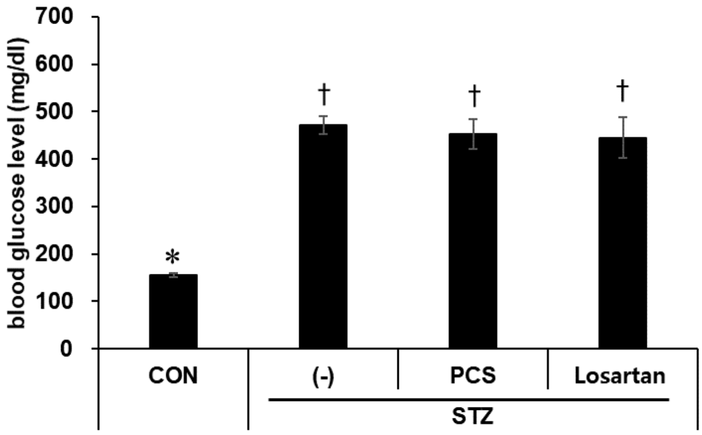

Fig. S1. Blood glucose levels in STZ-induced diabetic mice treated with PCS extract. Mice were injected with vehicle (CON) or STZ (50 mg/kg/day) for 5 consequtive days. STZ-induced diabetic mice (blood glucose levels > 300 mg/dl) were treated with vehicle (-), PCS extract (500 mg/kg/day) or losartan potassium (10 mg/kg/day) as a positive control for 8 weeks (n= 8-11/group) and blood glucose levels were determined. †, p<0.05 vs. CON mice.*, p<0.05 vs. (-)/STZ mice.

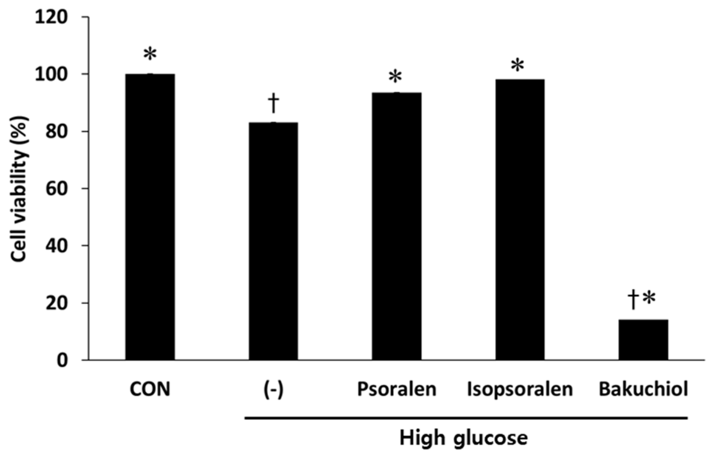

Fig. S2. Cell viability in SV40 MES-13 cells treated with 4 μg/ml of psoralen, isopsoralen or bakuchiol. SV40 MES-13 cells were treated with 4 μg/ml of psoralen, isopsoralen or bakuchiol for 24 h in the presence of high glucose concentration (25 mM). Cell viability was determined by CCK8 assay. †, $p<0.05$ vs. CON. *, $p<0.05$ vs. (-)/High glucose.
